# A New Animal Model for Investigation of Mechanical Unloading in Hypertrophic and Failing Hearts: Combination of Transverse Aortic Constriction and Heterotopic Heart Transplantation

**DOI:** 10.1371/journal.pone.0148259

**Published:** 2016-02-03

**Authors:** Andreas Schaefer, Yvonne Schneeberger, Justus Stenzig, Daniel Biermann, Marisa Jelinek, Hermann Reichenspurner, Thomas Eschenhagen, Heimo Ehmke, Alexander P. Schwoerer

**Affiliations:** 1 Department of Cardiovascular Surgery, University Heart Center Hamburg, Hamburg, Germany; 2 DZHK (German Center for Cardiovascular Research), Partner site Hamburg/Kiel/Lübeck, Hamburg, Germany; 3 Department of Experimental Pharmacology and Toxicology, University Medical Center Hamburg-Eppendorf, Hamburg, Germany; 4 Department of Cellular and Integrative Physiology, Center for Experimental Medicine, University Medical Center Hamburg-Eppendorf, Hamburg, Germany; Emory University, UNITED STATES

## Abstract

**Objectives:**

Previous small animal models for simulation of mechanical unloading are solely performed in healthy or infarcted hearts, not representing the pathophysiology of hypertrophic and dilated hearts emerging in heart failure patients. In this article, we present a new and economic small animal model to investigate mechanical unloading in hypertrophic and failing hearts: the combination of transverse aortic constriction (TAC) and heterotopic heart transplantation (hHTx) in rats.

**Methods:**

To induce cardiac hypertrophy and failure in rat hearts, three-week old rats underwent TAC procedure. Three and six weeks after TAC, hHTx with hypertrophic and failing hearts in Lewis rats was performed to induce mechanical unloading. After 14 days of mechanical unloading animals were euthanatized and grafts were explanted for further investigations.

**Results:**

50 TAC procedures were performed with a survival of 92% (46/50). When compared to healthy rats left ventricular surface decreased to 5.8±1.0 mm² (vs. 9.6± 2.4 mm²) (p = 0.001) after three weeks with a fractional shortening (FS) of 23.7± 4.3% vs. 28.2± 1.5% (p = 0.01). Six weeks later, systolic function decreased to 17.1± 3.2% vs. 28.2± 1.5% (p = 0.0001) and left ventricular inner surface increased to 19.9±1.1 mm² (p = 0.0001). Intraoperative graft survival during hHTx was 80% with 46 performed procedures (37/46). All transplanted organs survived two weeks of mechanical unloading.

**Discussion:**

Combination of TAC and hHTx in rats offers an economic and reproducible small animal model enabling serial examination of mechanical unloading in a truly hypertrophic and failing heart, representing the typical pressure overloaded and dilated LV, occurring in patients with moderate to severe heart failure.

## Background

Heart failure is the most common reason for hospital admission in the United States and Western Europe and is therefore connected with a major socio-economic burden [1, 2]. Due to global organ shortage and groundbreaking developments in mechanical circulatory support, the number of heart transplantations declined and the number of implanted left ventricular assist devices (LVAD) markedly increased over the last decade [3–5]. Since 2010, 100% of patients in need for destination therapy, registered in the Interagency Registry for Mechanically Assisted Circulatory Support (INTERMACS), received continuous-flow (CF) pumps. Miniaturization and durability of these innovative CF pumps improved outcome of patients with end-stage heart failure in terms of symptoms, hospitalization and premature death while awaiting transplantation [6]. Nonetheless, CF and mechanical unloading are non-physiological conditions for the myocardium and vessels. In experimental models of unloading severe alterations occur in micro ribonucleic acid expression, in cardiac electrophysiology, in the phosphorylation of cardiac regulatory proteins and cardiac atrophy [7–10]. Clinically it frequently leads to gastrointestinal bleeding due to arteriovenous malformation [11]. To what extent myocardial changes and cardiocytological alterations are beneficial or detrimental in the diseased heart is largely unknown.

Previous models to investigate mechanical unloading include large animals (bovine, canine, porcine) and small animal models (rat, mouse), in which heterotopic heart transplantation (hHTx) is performed [12, 13]. While large animal models are expensive and time-consuming, investigation of mechanical unloading in the hypertrophic and failing heart is possible by creating pressure overload by aortic banding or volume overload by artificial mitral regurgitation. On the other hand small animal models are advantageous regarding expenses and handling, whereby hHTx in mice, compared to rat models, put high requirements on microsurgical capabilities. Furthermore, studies of mechanical unloading to date are only performed in healthy or infarcted hearts, not representing the typical dilated heart of end-stage heart failure patients [14, 15].

In this article we present a new and economic small animal model to investigate mechanical unloading in hypertrophic and failing hearts: the combination of transverse aortic constriction (TAC) and hHTx in rats.

## Methods

All experiments were conducted in accordance to local institutional guidelines after approval from local authorities (Institutional Animal Care and Use Committee: Behörde für Gesundheit und Verbraucherschutz, Hamburg, Germany, File reference: G82/14). All animals included in the experiment received human care in compliance with the “Principles of Laboratory Animal Care” formulated by the National Society for Medical Research and the “Guide for the Care and Use of Laboratory Animals” prepared by the Institute of Laboratory Animal Ressources. Biometrical planning was used to reduce animal numbers. Buprenorphin and Carprofen were used to ameliorate suffering. Sacrification was conducted via an overdose of Pentobarbital and subsequent decapitation.

### TAC procedure

To induce cardiac hypertrophy and failure in rat hearts, three week old (40–50 g) male syngeneic Lewis rats (Charles River Germany GmbH, Sulzfeld, GER) underwent TAC procedure as follows:

Animals were anaesthetized by 2%-Isoflurane inhalation, intubated and ventilated with 100% oxygen (0.7 ml/100 g bodyweight [BW], 90/min) and cardiac hypertrophy was induced as described from Zaha and colleagues [16]. In summary, animals were placed in a supine position, a 2 cm median cut along the neck was performed and the underlying sternum was revealed. Then a 1.5 cm median hemisternotomy was performed, the sternum halves were kept apart with 4–0 Vicryl sutures^®^ (Ethicon Inc., Somerville, NJ, USA) which were later-on used to close the sternum. The prominent thymus was carefully put away to preserve the underlying structures. After dissection of the aorta from the pulmonary trunk a titanium clip was placed between the brachiocephalic trunk (TBC) and the left common carotid artery around the aortic arch. The clip was delivered by a clip applicator (WECK^®^, Horizon^™^Metal Ligation Sytem, Teleflex, Morrisville, NC, USA) which was provided with an adjustable screw, so that an internal diameter of 0.45 mm was left. After hemostasis was ensured the sternum and the skin were closed and the animals were kept on a heating plate until full consciousness was reached.

**Fig 1:**
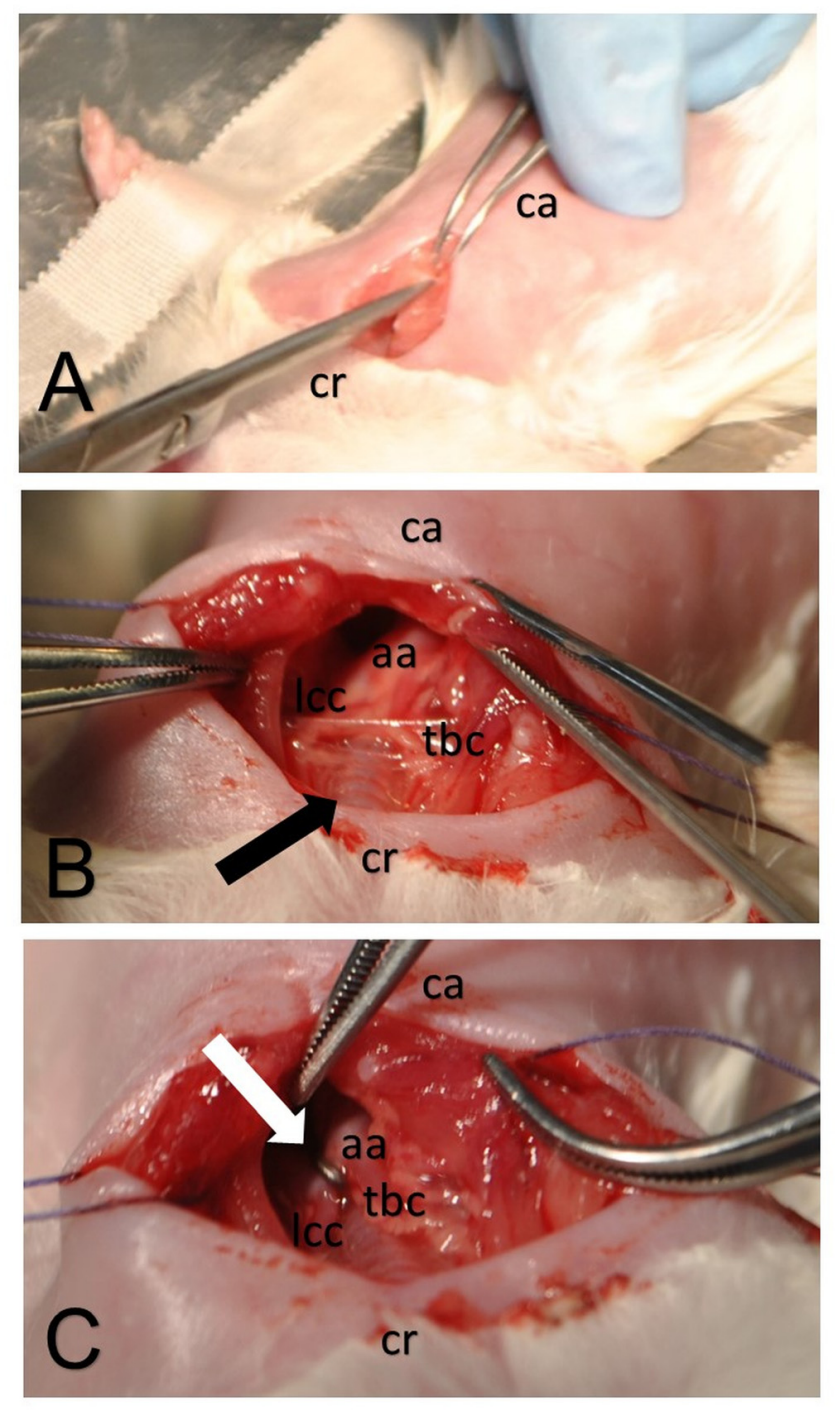
Surgical steps for transverse aortic constriction in three-week old Lewis rats. Upper hemisternotomy for access to aortic arch is performed with scissor from cranial (cr) to caudal (ca) (A). Brachiocephalic trunc (tbc) exits ascending aorta (aa) before trachea (black marker) is crossed and left common carotid (lcc) after trachea is crossed (B). Clip (white marker) is placed between tbc and lcc (C).

For depicted surgical steps of TAC procedure see Fig 1.

### Heterotopic heart transplantation

Two weeks after the initial TAC surgery, all animals were examined echocardiographycally (Vevo 770 system, 20 MHz center frequency single element transducer, VisualSonics Inc., Toronto, Canada). The systolic pressure gradient over the stenosis was evaluated by colour duplex sonography and only animals with a maximum gradient above 50 mmHg were included in the study. Three weeks after the procedure animals showed myocardial hypertrophy with preserved fractional shortening (FS) and six weeks after TAC thinned myocardium with reduced FS, as confirmed by echocardiography and histological examination, respectively.

**Fig 2:**
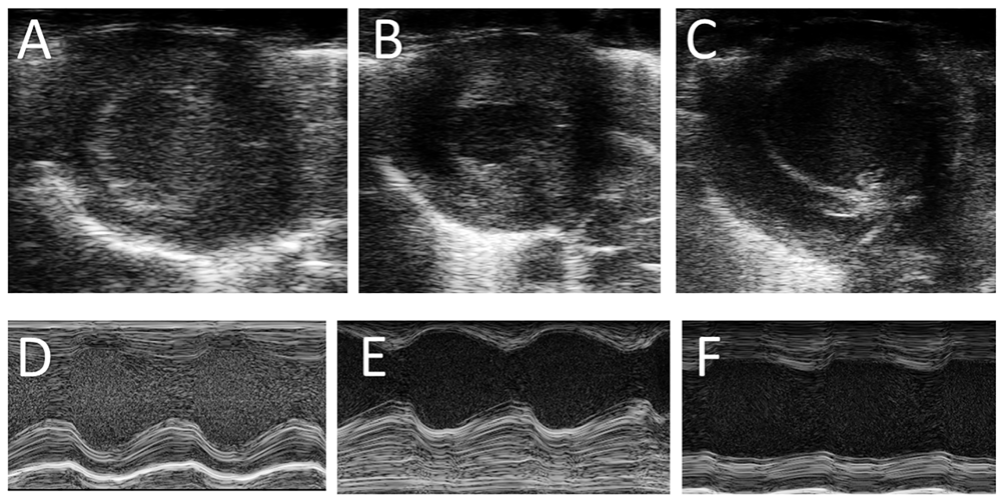
Echocardiography of healthy and pressure-overloaded rat hearts. Baseline echocardiography of healthy rat heart in B-Mode (A) and M-Mode (D). Rat heart 3 weeks after TAC with thickened myocardium and reduced end-systolic left ventricular diameter in B-Mode (B) and M-Mode (E). Rat heart 6 weeks after TAC with thinned myocardium and increased end-systolic left ventricular diameter in B-Mode (C) and M-Mode (F). Representative images.

**Fig 3:**
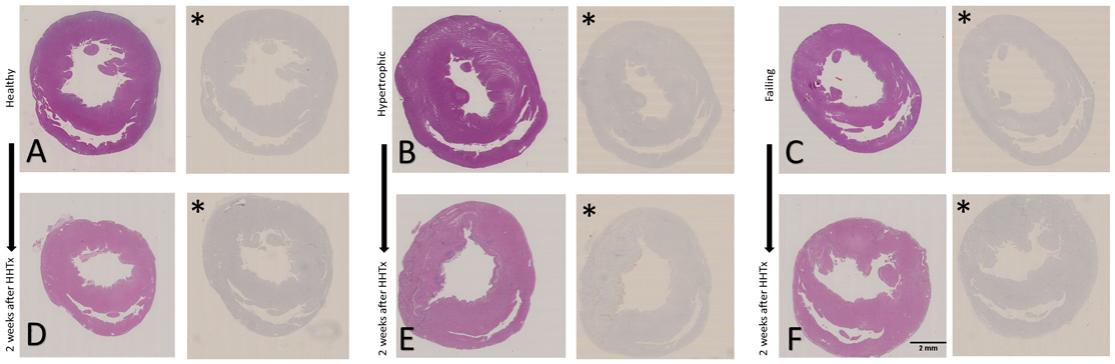
Left and right ventricular heart histology in transversal orientation and haematoxylin staining, Caspase-3 staining for evaluation of apoptotic myocyte numbers. A: Healthy heart of 3-weeks old Lewis rat B: Hypertrophic heart of 4-weeks old Lewis rat, 3 weeks after TAC with thickened myocardium C: Failing heart of 7-weeks old Lewis rat, 6 weeks after TAC with thinned myocardium D-F: Healthy heart (D), hypertrophic heart (E) and failing heart (F) of Lewis rat, after 2 weeks of mechanical unloading. * Caspase-3 staining of the respective image to the left. Representative images.

For exemplary depicted echocardiography and rat heart histology see Figs 2 and 3.

Subsequent to confirmation of restricted heart function, hHTx with hypertrophic and failing hearts in 220–250 g Lewis rats was performed to induce mechanical unloading. To reduce myocardial burden in the pre-impaired heart, donor and recipient animals were anesthesized with Sevoflurane (SEVOrane^®^, Abbott Laboratories Inc., Chicago, IL, USA) as it is considered to improve myocardial inotropy after reperfusion [17]. Analgesia was administered using 0.04 mg/kg BW buprenorphine (Temgesic^®^, Reckitt Benckiser, Slough, Berkshire, UK) and 4–5 mg/kg BW Carprofen s.c. in both, donor and recipient rat, and after opening of abdomen 500 I.E. Heparin was injected into the inferior vena cava (VCI) of the donor rat. Then, the chest was opened in a butterfly fashion with median sternotomy and cutting at the height of the diaphragm arch. Due to prior TAC, severe adhesions had to be detached after removement of the thymus. Especially ascending aorta (AA), pulmonal artery (PA), superior vena cava (VCS) and TBC tended to present fibrous aggregrations which had to be exceedingly careful dissected to preserve AA and PA for anastomotic capability and TBC for administration of cardioplegia. In few animals the clip grasped not only the AA but also the cranial wall of the PA. In this particular case the PA can be carefully pulled out of the clip or, if not possible, has to be later on cut at the proximal side of the clip.

Subsequent to topical cooling with ice-cold saline, rapid administration of cardioplegia (St. Thomas-Hospital solution I, Dr. Franz Köhler Chemie GmbH, Bensheim, GER) via the opened TBC is of crucial importance to protect the pre-damaged heart against further myocardial ischemia.

**Fig 4:**
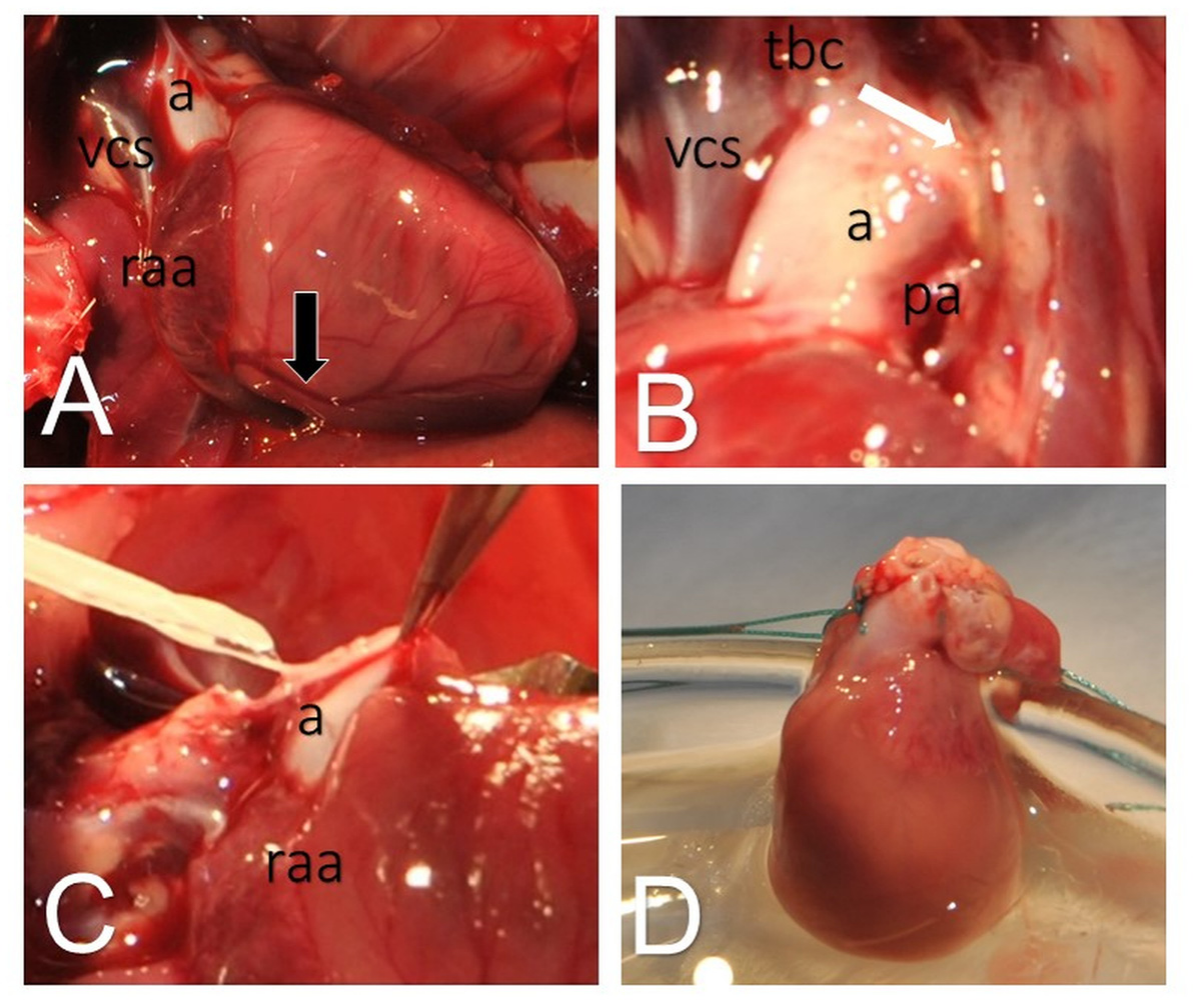
Technical considerations for heterotopic heart transplantation in Lewis rats with prior transverse aortic constriction. Exposition of hypertrophic heart three weeks after transverse aortic constriction with right coronary artery (black marker), aorta (a), superior vena cava (vcs) and right atrial appendage (raa) (A). View from anterior with aorta (a), clip (white marker) and adhesions of aorta, pulmonal artery (pa), superior vena cava (vcs) and brachiocephalic trunc (tbc) (B). Cardioplegia has to be administered rapidly (C) via a 22 Gauge (0.9 mm) canula leading to collapse of heart function and fading of coronary arteries (D).

See Fig 4 for adhesions and administration of cardioplegia during hHTx.

After loss of contractility and collapse of coronary arteries, VCS and VCI as well as pulmonary veins were ligated using Mersiline^®^ (Ethicon Inc., Somerville, NJ, USA). As described by Wang and colleagues a coronary branch running along the AA was ligated [18] and AA and PA were cut. Compared to hHTx with healthy grafts, AA and PA were significantly shorter due to adhesions and prior applied clip of the TAC procedure, resulting in a more challenging anastomosing to the recipient vessels.

Transplantation then was performed in classical way, as described by Ono and Lindsey [19], consisting of anastomosing the AA to the recipient’s abdominal aorta and the PA to the abdominal part of the recipient’s VCI using Prolene 8–0 (Ethicon Inc., Somerville, NJ, USA).

Due to the syngeneic nature of the utilized Lewis rats no immunosuppression had to be performed. After 14 days of mechanical unloading, with daily digital testing of graft contractility, animals were euthanatized and grafts were explanted for further investigations.

### Histology and immunohistochemistry

For both histology (Picro-Sirius Red staining) and immunohistochemistry (dystrophin and Caspase-3 staining) rat hearts were formaldehyde fixed for 24 h. After paraffin embedding 3 μm sections were cut strictly transversally for Picro-Sirius Red staining or strictly longitudinally for immunohistochemistry. Picro-Sirius Red staining was performed in a fully automated manner. For immunohiystochemistry optimized staining conditions were: rat anti-dystrophin monoclonal antibody (Millipore, MAB1645), dilution 1:200, antigen retrieval: 60 min in EDTA-buffer, pH 8.0. Dystrophin immunohistochemistry was visualized using the multimer-technology based UltraView Universal DAB Detection kit. For image quantification, Image J software was used by a blinded investigator. Degree of fibrosis was assessed by area quantification on Picro-Sirius Red stained transversal sections, cell diameter was quantified by manual measurement of maximal diameter (at right angles with cell axis) of at least 40 cells per heart on dystrophin stained longitudinal sections.

### Statistics

Continuous variables are presented as mean ± standard deviation and categorical variables are presented as percentage. Comparisons of continuous variables were made with paired t-test.

## Results

Animals presented significant hypertrophy and decreased systolic function 3 and 6 weeks after TAC procedure, respectively (see Figs 2 and 3, see Table 1). Stenosis was validated utilizing echocardiography with a max/mean pressure gradient of 60/20±17/6 mmHg one week after intervention. When compared to healthy rats left ventricular surface decreased to 5.8±1.0 mm² (vs. 9.6± 2.4 mm²) (p = 0.001) after three weeks of pressure overload with a fractional shortening (FS) of 23.7±4.3% vs. 28.2±1.5% (p = 0.01). Six weeks after application of the clip, systolic function decreased to 17.1±3.2% vs. 28.2±1.5% (p = 0.0001) and left ventricular inner surface increased to 19.9±1.1 mm² (p = 0.0001). Furthermore, animals presented clinical signs of heart failure such as dyspnea, rough fur and pale-bluish limbs six weeks after intervention.

**Table 1:** Echocardiographic parameters.

	Healthy	Hypertrophed	Failing	Healthy hHTx	Hypertrophed hHTx	Failing hHTx
**Echocardiography**						
** Ejection fraction (%)**	52.1±2.3	33.8±4.5	20.5±16.1	/	/	/
p-value*	/	0.0001	0.0001	/	/	/
** Fractional shortening (%)**	28.2±1.5	23.7±4.3	17.1±3.2	/	/	/
** p-value**	/	0.01	0.0001	/	/	/
** Wall thickness (mm)**	1.9±0.1	2.2±0.3	2.1±0.2	/	/	/
** p-value**	/	0.02	0.02	/	/	/
** Left ventricular surface (mm²)**	9.6± 2.4	5.8±1.0	19.9±1.1	/	/	/
** p-value**	/	0.001	0.0001	/	/	/
** Enddiastolic volume (μl)**	580.1±19.5	1407.7±250	1596.4±211.7	/	/	/
** p-value**	/	0.0001	0.0001	/	/	/
** Endsystolic volume (μl)**	278.3±18.5	982.6±135.8	1287.9±388	/	/	/
** p-value**	/	0.0001	0.0001	/	/	/
**Histology**						
** Myocyte cell size (μm)**	34.3±5.9	44.9±3.1	49.8±3.1	30.7±6.6	39.8±3.9	38.7±6.4
** p-value**^#^	/	/	/	0.27	0.01	0.0006
** Connective tissue area (pixel no.)**	2642±288	5607±3201	5653±7715	216712±16567	185196±14671	151631±82573
** p-value**	/	/	/	0.0001	0.0001	0.0002

hHTx- heterotopic heart transplantation;

*p-values of echocardiography section computed with values of healthy group;

^#^ p-values of histology section computed with values of according group (healthy compared to healthy hHTx etc.)

Regarding heart mass per tibia length, healthy rats presented a mean ratio of 27.8±0.96 mg/mm vs. 37.1±6.2 mg/mm in hypertrophed rat hearts (p = 0.025) and 54.6±9.2 mg/mm in failing rat hearts (p = 0.01 for healthy rat hearts; p = 0.02 for hypertrophed rat hearts). In Caspase-3 staining no impressive differences between the groups regarding apoptotic myocyte cell numbers were found (see Fig 3).

Overall 50 TAC procedures were performed with a survival until scheduled hHTx of 92% (46/50). Two animals died due to intraoperative bleeding and two died due to heart failure. Intraoperative graft survival during hHTx was 80% with 46 performed procedures (37/46). Of nine graft losses, six have to be attributed to the early phase of establishment of this new model with intraoperative tearing of AA and PA during dissection of adhesions. Three more losses included bleeding complications during preparation of the abdominal vessels of the recipient rat. Due to the low ischemia tolerance of pre-damaged hearts, these organs had to be discarded.

All transplanted organs survived two weeks of mechanical unloading in the recipient abdomen as shown by continuous and regular beating verified by palpation. After two weeks of mechanical unloading all grafts presented lower weight. The mean loss amounted to 0.2 g in healthy, 0.35 g in hypertrophic and 1.2 g in failing hearts. Histology (see Fig 3) showed typical signs of atrophic myocardial processes such as fibrosis and change in myocyte sizes (see Table 1).

When compared to hHTx of healthy hearts at our institution, initial graft and recipient survival was lower during the initial phase of this model. Nevertheless it was increased to the same level after several weeks of practicing with identification of the major challenges: careful dissection of adhesions surrounding the graft and rapid cooling of the donor heart and administration of cardioplegia to reduce myocardial ischemia to a minimum.

## Discussion

Steady flow MCS found widespread acceptance for the therapy of end-stage heart failure over the last decade. Despite obvious advantages in clinical daily routine, there is a lack of knowledge concerning mechanisms leading to myocardial changes and cardiocytological alterations under mechanical unloading of failing hearts. To date small animal models solely investigate mechanical unloading in healthy or infarcted hearts.

With the herein presented combination of TAC and hHTx the authors offer a quick, economic and reproducible small animal model enabling serial examination of mechanical unloading in large animal numbers. Major advantage of the introduced model is the imitation of MCS in genuinely hypertrophic and failing hearts, representing the typical pressure overloaded and dilated LV, occurring in patients with moderate to severe heart failure. By means of echocardiography and histology, heart hypertrophy and heart failure were verified three and six weeks subsequent to TAC, respectively. Compared to Shingu and colleagues, who firstly showed echocardiographic reproducible determination of heart failure stages after aortic constriction in rats and reported a reduction of FS to occur 10 weeks after TAC, we observed a significant reduction of FS already six weeks after TAC [20]. Most likely, this is caused by differences in the degree of aortic constriction. This observed variance actually offers the prospective possibility for sophisticated investigation of mechanical unloading in different graduation of pressure overloaded hearts and consecutive heart failure.

HHTx after TAC is technically demanding when compared to hHTx of healthy hearts. However, once dissection of adhesions is sufficiently trained and ischemia time is minimized graft survival approaches success rate of hHTx utilizing healthy rat hearts. Nevertheless, routine digital palpation of abdominal located grafts is mandatory to ensure enduring graft survival. In our institution grafts are explanted 14 days after hHTx based on the assumption that long term excessive mechanical unloading may adversely affect the process of cardiac reverse remodeling.

In conclusion, the presented model enables cost- and time-effective clinical and basic research of mechanical unloading in a large number of animals in a truly hypertrophic and failing heart, representing end-stage heart failure.

## Abbreviations

AA: Ascending aorta
BW: bodyweight
CF: Continuous-flow
FS: Fractional shortening
hHTx: Heterotopic heart transplantation
INTERMACS: Interagency Registry for Mechanically Assisted Circulatory Support
LV: Left ventricle/ventricular
LVAD: Left ventricular assist device
MCS: Mechanical circulatory support
PA: Pulmonal artery
TAC: Transverse aortic constriction
TCB: Brachiocephalic trunk
VCI: Vena cava inferior
VCS: Vena cava superior
